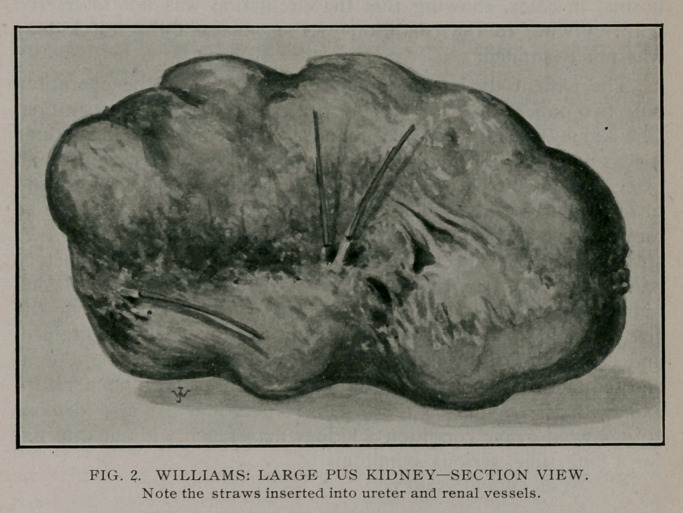# A Case of Large Pus Kidney, with Exhibition of Specimen1Specimen exhibited at a meeting of the Hospital Medical Society of Rochester, N. Y.

**Published:** 1906-10

**Authors:** Henry T. Williams

**Affiliations:** Rochester, N. Y.; Surgeon to Rochester City Hospital and Saint Mary’s Hospital


### CLINICAL REPORT.

### A Case of Large Pus Kidney, with Exhibition of Specimen.[1]

By HENRY T. WILLIAMS, M. D., Rochester, N. Y.
Surgeon to Rochester City Hospital and Saint Mary's Hospital.

MRS. A. A. F———, widow; age 75 years. Always has been in usually good health except occasional colds and indigestion, but no serious illness. I first saw her April 6, 1906. She had been ill for several days suffering from a hard cold in her head and pain in the bowels, mostly in right side of abdomen, and some vomiting. When she came under my observa-

FIG. 1. WILLIAMS: LARGE PUS KIDNEY—FRONT VIEW.

tion her temperature under the tongue was 102° F.; had been able to take but little nourishment on account of pain in abdomen and nausea and occasional vomiting. The abdomen was sore and very tender upon pressure, especially over the McBurney point area where a large mass could be felt extending slightly beyond the median line and into the loins on the right side. Dulness on percussion and elastic and fluctuating were marked, more so over the McBurney point. The next day, April 7, dulness and tenderness had extended over a larger area and was more elastic and fluctuating. The urine, 1022 sp. gr., contained a trace of albumin, but no casts and no pus. Temperature 101 4-5; bowels had moved freely; nausea and vomiting increased.

1. Specimen exhibited at a meeting of the Hospital Medical Society of Rochester, N. Y.

I operated upon her that night. While under the anesthetic a large irregular shaped mass could be felt extending below the liver down to the lower part of the abdomen, into the right loins and back and beyond the median line of the abdomen, the most prominent part over McBurney point. The diagnosis was, tumor of the right kidney. A large median incision was made extending from three inches above pubes to three inches above the navel. The capsule was found to be very vascular, and several ligatures and hemostats were applied to stop the hemorrhage. It was noticed that the vessels were very much enlarged and sclerotic, (even calcareous), breaking frequently under the for-

FIG. 2. WILLIAMS: LARGE PUS KIDNEY—SECTION VIEW.

Note the straws inserted into ureter and renal vessels.

ceps and ligatures. The ureter was quite large and was tied with two ligatures and cut between them.

After the capsule had been divided the kidney was easily drawn out and the renal vessels exposed. These were tied with catgut ligatures but the vessels were so hard and brittle that they several times broke off, and finally were tied in mass after clamping them together with some of the surrounding tissue. While carefully lifting out the large kidney there was a sudden gush of dark blood. Dr. Boddy, who was assisting me, put his hand under the tumor and grasped the venacava which stopped the hemorrhage. The kidney was then rapidly cut off at its pedicle (where the ligature had been applied to the vessels) and removed. It was found that the renal vein had torn off from the vena cava; both venacava and aorta were felt to be very hard and inelastic and seemed to be studded with calcarious deposits.

The tear in the venacava was grasped with forceps, but owing to its brittle condition, tore open again. A compress of sterile gauze was placed against the opening and other gauze compresses against that and strong strips of oxide of zinc plaster across the upper part of the abdomen holding them in place, whereupon the hemorrhage ceased. The rest of the abdominal incision was brought together with silkworm gut sutures. The patient rallied from the operation. Pulsation of the arteries of the feet could be plainly felt and the feet and lower extremities were normal in color, showing that the circulation was not interfered with. Owing to the condition of the venacava the left kidney was not examined.

The patient died from uremia 48 hours after the operation, only two ounces of urine passing per catheter after the operation. The kidney weighed twelve pounds. Upon opening the kidney it was found to be filled with a thick creamy pus, sacculated in places on outer part of kidney where the pus was gelatinous and in some places caked. The outer shell of the kidney as it may be termed, or pus sac, was only about 1-40 of an inch thick. A straw carried through the ureter and one through the renal artery passed directly into the pus sac. No evidence of any normal kidney substance remained. The case is interesting from the size of the kidney and from the amount of pus which it contained, and because of the thinning of its walls, the age of the patient and the fact that no serious symptoms or pain or high temperature existed until so short a time before operation. It also demonstrates what grave condition a kidney may reach without causing, for a long time, serious symptoms.

274 Alexander Street.

## Figures and Tables

**Fig. 1. f1:**
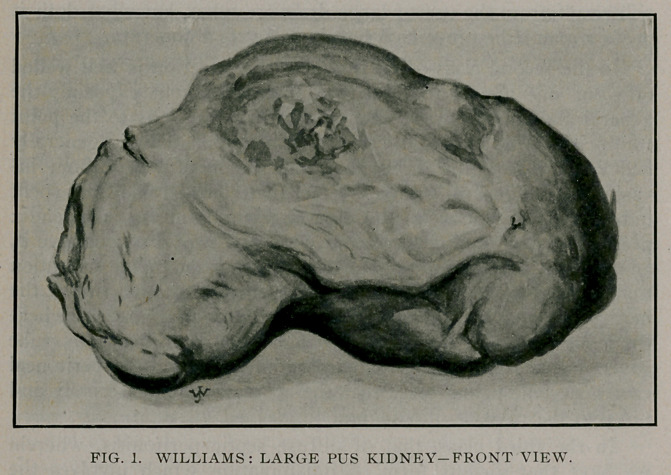


**Fig. 2. f2:**